# Thermal Stability and Decomposition Products of P-Doped Ferrihydrite

**DOI:** 10.3390/ma13184113

**Published:** 2020-09-16

**Authors:** Gabriela Pieczara, Maciej Manecki, Grzegorz Rzepa, Olaf Borkiewicz, Adam Gaweł

**Affiliations:** 1 Faculty of Geology, Geophysics and Environmental Protection, AGH-University of Science and Technology, al. Mickiewicza 30, 30-059 Kraków, Poland; gabriela.pieczara@gmail.com (G.P.); gprzepa@cyf-kr.edu.pl (G.R.); agawel@agh.edu.pl (A.G.); 2 X-ray Science Division, Advanced Photon Source, Argonne National Laboratory, Argonne, IL 60439, USA; borkiewicz@aps.anl.gov

**Keywords:** P-ferrihydrite, rodolicoite, grattarolaite, hematite, maghemite, Fe-phosphate, thermal transformations

## Abstract

This work aimed to determine the effect of various amounts of P admixtures in synthetic ferrihydrite on its thermal stability, transformation processes, and the properties of the products, at a broad range of temperatures up to 1000 °C. A detailed study was conducted using a series of synthetic ferrihydrites Fe_5_HO_8_·4H_2_O doped with phosphates at P/Fe molar ratios of 0.2, 0.5, and 1.0. Ferrihydrite was synthesized by a reaction of Fe_2_(SO_4_)_3_ with 1 M KOH at room temperature in the presence of K_2_HPO_4_ at pH 8.2. The products of the synthesis and the products of heating were characterized at various stages of transformation by using differential thermal analysis accompanied with X-ray diffraction, Fourier transform infrared spectroscopy, and scanning electron microscopy-energy dispersive X-ray spectroscopy. Coprecipitation of P with ferrihydrite results in the formation of P-doped 2-line ferrihydrite. A high P content reduces crystallinity. Phosphate significantly inhibits the thermal transformation processes. The temperature of thermal transformation increases from below 550 to 710–750 °C. Formation of intermediate maghemite and Fe-phosphates, is observed. The product of heating up to 1000 °C contains hematite associated with rodolicoite FePO_4_ and grattarolaite Fe_3_PO_7_. Higher P content greatly increases the thermal stability and transformation temperature of rodolicoite as well.

## 1. Introduction

Ferrihydrite (Fe_5_HO_8_∙4H_2_O) is a widely known hydrous ferric oxyhydroxide that is widespread in both terrestrial and aquatic systems [1]. It is formed in near-surface environments during the oxidation reaction of Fe(II) to Fe(III), as a nearly amorphous nanomineral with a high surface area [2,3,4,5], and a high concentration of active hydroxyl sites [6]. It plays an essential role as a sorbent of various major and trace elements and controls their availability in the environment [7,8,9,10,11]. Therefore, natural ferrihydrite always contains a variety of impurities that affect its chemical and physical properties [12,13,14,15,16,17,18,19]. Some impurities such as phosphate are important nutrients, while other impurities may be unwanted compounds, toxic metals and metalloids (e.g., Cd, Pb, As). Their occurrence and speciation are strongly dependent on the stability of ferrihydrite under environmental conditions [20,21,22]. Although pure ferrihydrite is unstable and directly transforms to more stable phases such as goethite or hematite [2], many recent studies have proved that the admixtures present in ferrihydrite significantly inhibit its transformation [23,24]. In the presence of phosphate, the solubility of ferrihydrite decreases [25,26]. Consequently, the rate of ferrihydrite conversion is reduced [27] and the aggregation of colloidal iron oxide particles is observed [28,29] which leads to the preferred formation of hematite [27,30]. Arsenate also leads to the aggregation of ferrihydrite particles, decreases the transformation rate, and promotes the formation of hematite over goethite [27]. Feely et al. [31] demonstrated a strong positive relationship between the molar P/Fe ratio in fresh hydrothermal precipitates and seawater-dissolved phosphate, thus indicating the scavenging of dissolved phosphate from seawater during the precipitation of hydrothermal Fe-rich particles above hydrothermal vents.

The two principal mechanisms by which iron(III) (oxy)hydroxide transforms into secondary phases are hydrothermal transformations through a dissolution-reprecipitation mechanism and thermal transformation in the absence of the solution [2]. Thermal transformation processes are controlled by many factors, such as temperature, atmosphere, presence of impurities, and substitutions, all of which affect the reaction rate and mechanism. Previous studies have shown that iron(III) oxyhydroxides transform thermally into hematite [32,33]. This transformation path is strongly affected by the presence of phosphate adsorbed onto ferrihydrite during or after its precipitation [34]. Therefore, the present study aimed to quantify how the phosphate content affects the thermal pathway of ferrihydrite-hematite transformations and the potential formation of intermediate phases. Under certain conditions, P-doped ferrihydrite may undergo thermal conversion to hematite α-Fe_2_O_3_ through various intermediate phases (including maghemite γ-Fe_2_O_3_) and iron phosphates, including rodolicoite FePO_4_ and grattarolaite Fe_3_PO_7_ (or FePO_4_O_3_) [35].

Rodolicoite and grattarolaite are rare anhydrous iron(III) phosphates that may form as products of thermal conversion of amorphous hydrated iron(III) phosphates. The formation of these minerals is attributed to the thermal conversion and oxidation of iron phosphates, particularly vivianite Fe^2+^_3_(PO_4_)_2_·8H_2_O, resulting from the combustion of mine waste in coal mines or ore deposits that contain pyrite FeS_2_, iron phosphates and carbonaceous materials [35]. The authors of [35] suggest that combusted mine waste materials may have reached temperatures of at least 1000 °C. Spontaneous combustion results in the production of heat (>270 °C near the surface and up to 1200 °C deep in a waste rock dump) which affects, among other things, the formation of various mineral species. In natural environments, however, the path of formation of these phosphate minerals is extremely difficult to reconstruct. This can be accomplished with the thermal analysis of synthetic metastable ferrihydrite Fe_5_HO_8_∙4H_2_O doped with phosphates.

To date, the effect of coprecipitated P on thermal properties and the transformation path of poorly crystalline iron minerals has been seldom investigated. A recent study [36] indicates that trace amounts of P in ferrihydrite do not alter the path of thermal transformation of ferrihydrite, which converts directly into hematite upon heating up to 700 °C. On the other hand, our study reveals the significant retardation of thermal transformation of ferrihydrite at the higher phosphate levels. Therefore, in this work, we present important insights concerning thermal stability and transformation mechanisms of a series of synthetic ferrihydrites Fe_5_HO_8_·4H_2_O, doped with phosphates at P/Fe molar ratios of 0.2, 0.5, and 1.0. This work aimed to determine the effect of various amounts of P admixtures in synthetic ferrihydrite on its thermal stability, transformation processes, and the properties of the products at a broad range of temperatures up to 1000 °C. For this purpose, a detailed study was conducted at various stages of transformation by using thermal analysis (STA-DTA/TG) accompanied with X-ray diffraction (XRD), Fourier transform infrared spectroscopy (FTIR), and scanning electron microscopy-energy dispersive X-ray spectroscopy (SEM-EDS).

## 2. Materials and Methods

### 2.1. Synthesis

**Figure 1:**
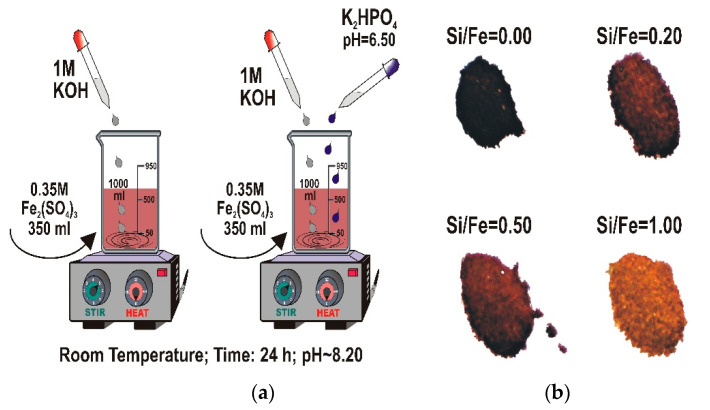
(**a**) Experimental setup for the synthesis of pure ferrihydrite and (**b**) photographs of ferrihydrite doped with P. The coprecipitation of P with ferrihydrite results in the brightening of the dry product.

A series of 2-line ferrihydrite samples with various P/Fe molar ratios of 0.0, 0.2, 0.5, and 1.0 (referred below as FHYD-0, FHYD-02, FHYD-05, and FHYD-1, respectively) was synthesized using a method analogous to previous studies on Si-ferrihydrites [24,37,38,39], by reaction of Fe_2_(SO_4_)_3_ with 1 M KOH at room temperature, in the presence of K_2_HPO_4_ at pH 8.2 (Figure 1). After 24 h of stirring, the samples were incubated for 4 days at room temperature and then dialyzed to remove the excess of salts. The dialysis was terminated when the electrical conductivity of the suspension decreased to below 3 μS cm^−1^. The ferrihydrite slurry was centrifuged and then stored dry after freeze-drying (Christ Alpha 1–2 LD apparatus, Osterode, Germany). Polyethylene vessels, double distilled water, and analytical grade reagents (supplied by Avantor Performance Materials, Poland) were used in all the experiments.

### 2.2. Thermal Transformation

The thermal transformation of P-doped ferrihydrites was conducted in the range of temperatures from 25 to 1000 °C. These temperatures are likely to occur in nature, e.g., in self-burning coal waste piles and ore deposits or wildfires [35,36,40]. Thermal transformation was studied by simultaneous thermal analysis–differential thermal analysis (STA-DTA) and thermogravimetry (TG). This was followed by heating experiments: each sample was individually heated in a furnace to the temperatures carefully chosen based on DTA/TG curves. The aliquots identical to those used for DTA were placed in a porcelain crucible and roasted at different temperatures in a muffle furnace (Czylok–FCF 26SH, Jastrzębie Zdrój, Poland). The heating was stopped as soon as the desired temperature was reached, and the sample was immediately removed from the oven and quenched in air. The samples attained temperatures below 100 °C within 1–3 min.

DTA and TG measurements were performed using a Netzsch STA 449F3 Jupiter instrument (Selb, Germany). A sample of ca. 50 mg was placed in an alumina crucible and heated from 20 to 1000 °C at the rate of 10 °C min^−1^ in the flow of synthetic air (40 mL min^−1^). A preheated sample was used as an inert material. Simultaneous analyses of the evolved gases were performed using a quadrupole mass spectrometer (Netzsch QMS 403C Aeolos, Selb, Germany). The thermal patterns were collected and processed using the Netzsch Proteus Thermal Analysis software (v. 6.0.0) Five reference substances, namely indium, tin, bismuth, aluminum, and gold, were used for temperature calibration. The precisions of DTA and TG measurements, estimated on the basis of repeated measurements of pure ferrihydrite, are <1 °C and <1 mass%, respectively.

### 2.3. Characterization of P-Doped Ferrihydrites

The samples were characterized prior to thermal transformation, at various stages of heating, and after final thermal transformation by using XRD, FTIR, and SEM-EDS. XRD patterns were collected using a Rigaku SmartLab instrument (Rigaku, Tokyo, Japan) equipped with a graphite monochromator, a rotation Cu anode, generator settings of 45 kV and 200 mA, recording range of 2°–75° 2Θ with 0.05° step size, and counting time of 1 s per step. The effect of instrumental parameters on peak intensity was minimized before each analysis, by adjusting the primary beam values with a plano-parallel plate made of highly crystalline quartz. The XRD patterns were evaluated by XRAYAN software (v. 4.0.5) using a diffraction pattern database of the International Center for Diffraction Data.

SEM analyses were performed in the low vacuum mode using an FEI 200 Quanta FEG microscope (FEI, Hillsboro, OR, USA) equipped with an EDS/EDAX spectrometer. The acceleration voltage and pressure were 15–20 kV and 60 Pa, respectively. The samples were not coated with a conductive layer.

Infrared spectra were acquired using Bruker Sensor 27 spectrometer (BRUKER, Ettlingen, Germany) in the range of 400–4000 cm^−1^ (64 scans at the resolution of 1 cm^−1^). Prior to analysis, KBr pellets were prepared by homogenizing 200 mg of ground KBr with 4 mg of the sample.

## 3. Results and Discussion

### 3.1. Properties of P-Doped Ferrihydrites

SEM analysis reveals that all syntheses resulted in a homogenous cryptocrystalline precipitate. The actual P/Fe molar ratios determined by multiple SEM/EDS microanalyses are close to those that were intended (data not shown). Additionally, a small amount of potassium K was detected in all samples containing phosphate, increasing with increasing P content (Figure S1). This contamination comes from K_2_HPO_4_, which was the source of PO_4_ in the synthesis.

**Figure 2:**
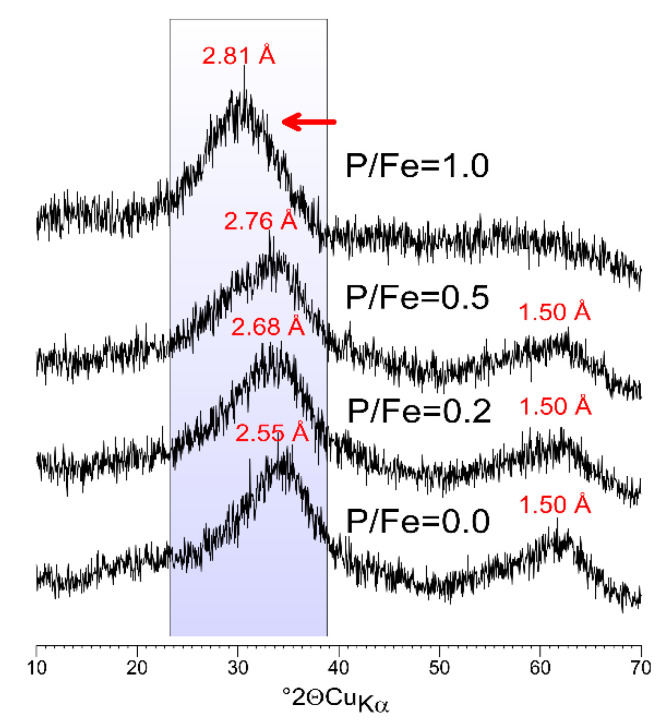
XRD patterns of a synthetic 2-line ferrihydrite doped with P (before heating). The increasing presence of P results in the systematic shift of the first peak toward the low angles. The second peak disappears at the highest content of P.

The XRD pattern of a pure, P-free sample is characterized by two broad peaks at d ~2.5 and 1.50 Å (Figure 2), which is typical for 2-line ferrihydrite [1,2,41,42]. Synthesis in the presence of P also resulted in the formation of 2-line ferrihydrite. These results agree well with previous works, revealing that PO_4_ inhibits crystal growth and reduces the crystallinity of iron oxyhydroxides at low P/Fe molar ratios [43], whereas a high P/Fe ratio results in the formation of amorphous FePO_4_ [44,45,46].

Increasing P content results in gradual broadening, decline of asymmetry, and shift of the first peak toward the lower 2Θ angles. Similar features were observed for ferrihydrites doped with Si, P and phytic acid [24,45,47]. This indicates a reduction of crystallinity and/or decrease in crystal size. More importantly, increasing the P content in FHYD-02 and FHYD-05 samples does not change the position of the second peak on the XRD pattern. At the highest P/Fe molar ratio, the second peak almost vanishes, thus indicating a loss of crystallinity and the formation of amorphous iron(III) phosphate [17,44]. These results agree well with previous works, revealing that PO_4_ inhibits crystal growth and reduces crystallinity of iron oxyhydroxides at low P/Fe molar ratios [43], whereas a high P/Fe ratio results in the formation of amorphous FePO_4_ [44,45,46]. Thibault et al. [44] indicated that the limiting P/Fe ratio in ferrihydrite is approximately 0.5, a value earlier reported as the maximum in natural samples [48]: at this P/Fe ratio, a characteristic two-line ferrihydrite X-ray pattern resembling our P/Fe = 0.5 sample was obtained. Any excess PO_4_ present in their synthesis solution of P/Fe = 0.75 and higher resulted in a virtually one-line XRD pattern characteristic of amorphous Fe phosphate, resembling our sample of P/F = 1.0.

**Figure 3:**
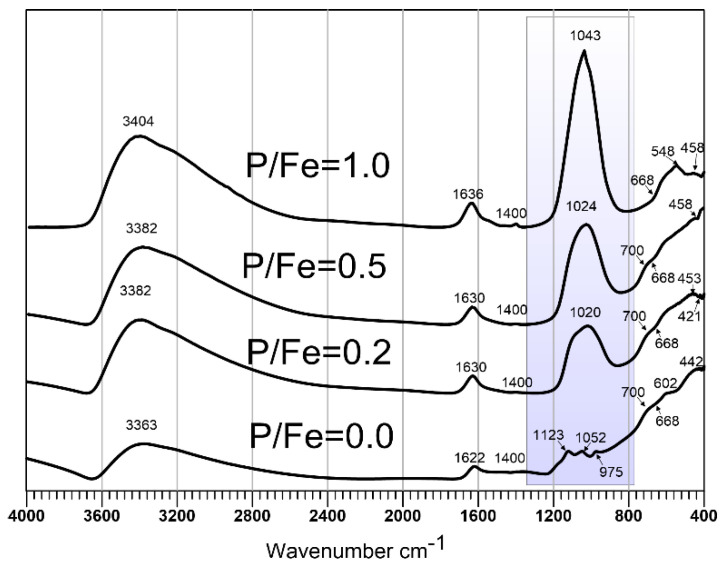
Background-corrected FTIR spectra of synthetic P-free and P-doped ferrihydrites (before heating). The bands in the shaded area indicate the presence of PO_4_^3−^. The position of this band shifts slightly toward higher wavenumbers with the increase in P content.

FTIR spectra of synthetic P-doped ferrihydrites are presented in Figure 3. For the full description of pure ferrihydrite spectra, see [24]. Briefly, the maxima at ca. 442 and 602 cm^−1^ result from stretching Fe–O vibrations, and intensive bands at ca. 3363 and 1622 cm^−1^ are assigned to the stretching and bending OH vibrations of the lattice and to the adsorbed water, respectively [2,47,49,50,51]. The bands at 974, 1050, and 1123 cm^−1^ are derived from sulfate–ferrihydrite complexes [52] and these bands can be associated with symmetric v1 and antisymmetric v3 stretching bands of SO4 tetrahedra [53,54]. This artifact resulting from the presence of residues of the reagents used in the synthesis was observed previously [24]. A very small, sharp peak at 668 cm^−1^ is attributed to small amounts of CO_2_ adsorbed from the air (antisymmetric stretching of the CO_2_ molecule) and is not an inherent feature of the samples.

The presence of P significantly affects infrared absorption spectra of ferrihydrite. The broad, undivided phosphate band in the range 900–1150 cm^−1^ is the most pronounced. This band results from P–O antisymmetric stretching mode ν_3_ [34,45,49,55] and overlaps with 1035 cm^−1^ band corresponding to stretching Fe–O–P bonds [51]. An identical wide phosphate band is observed on the infrared spectra of several iron-phosphate minerals, e.g., on the spectrum of rodolicoite Fe^3+^(PO_4_), or on the spectrum of santabarbaryite Fe_3_^3+^(PO_4_)_2_(OH)_3_∙H_2_O [35,56]. These bands are broader in the case of amorphous or poorly crystalline iron phosphates [51]. The position of the maximum of this band shifts with P from 1020 cm^−1^ for P/Fe = 0.2, through 1024 cm^−1^ for P/Fe = 0.5, and then to 1043 cm^−1^ for P/Fe = 1. This shift may reflect a variation (reduction) in bond strength. Additionally, this shift may be associated with the polymerization of phosphate tetrahedrons and formation of polyphosphates on the surface of ferrihydrite [57]. The position of the deformation band of H_2_O molecule at 1630–1636 cm^−1^ remains basically unchanged. In the range of 400–700 cm^−1^, the bands from P–O bonds and Fe–O bonds overlap. These characteristics of ferrihydrite (from stretching Fe–O vibrations) are only slightly shifted toward higher wavenumbers: from 445 to 458 cm^−1^, from 590 to 600 cm^−1^, and shoulder from 690 to 700 cm^−1^. Bands attributable to FePO_4_ and similar iron phosphates are present around 550 and 1070 cm^−1^ [49,58] In most of our spectra, the 1069 cm^−1^ band is included (and hidden) within a broad, general phosphate band. However, 548 cm^−1^ or a similar band can be distinguished on some occasions in the spectra of low temperature samples, thus confirming the formation of Fe–P bonds in P-doped ferrihydrite.

### 3.2. Thermal Analysis and Identification of the Products of Thermal Transformations

**Figure 4:**
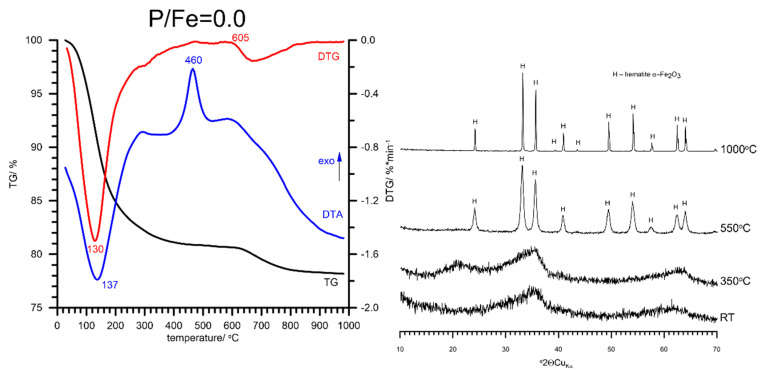
The results of thermal analysis and relevant XRD patterns of pure synthetic ferrihydrite and products of its thermal transformations at various temperatures. The formation of hematite (α-Fe_2_O_3_) at 460 °C, which remains stable up to 1000 °C, is apparent. No intermediate phase was detected. RT—room temperature.

The DTA-TG-DTG curves of FHYD-0 are presented in Figure 4. The DTA curve of FHYD-0 shows a broad endotherm with the maximum temperature at ca. 137 °C and a sharp exotherm at ca. 460 °C. The endotherm results from the removal of the surface H_2_O groups from ferrihydrite. The exotherm indicates the completion of the dehydration/dehydroxylation processes, which results in the crystallization of hematite according to the following simplified reaction:

2Fe_5_HO_8_∙4H_2_O → 5Fe_2_O_3_ + 5H_2_O

A total mass loss of FHYD-0 is equal to 21.28 wt %, and includes small mass loss (2.2 wt %) caused by the decomposition of sulfates. Small amounts of sulfates are present in the sample as remnants of the reactants used in the synthesis [24]. This is confirmed by a quadrupole mass spectrometry (QMS) signal, recording a release of SO_2_ between 600 and 800 °C (Figure S2).

**Figure 5:**
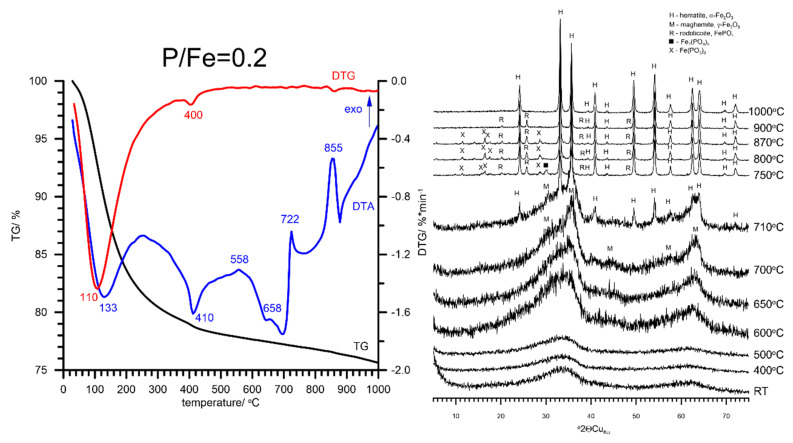
The results of thermal analysis and XRD patterns of P-doped synthetic ferrihydrite at low P/Fe molar ratio are equal to 0.2. Note the formation of nanomaghemite γ-Fe_2_O_3_ at around 700 °C and rodolicoite FePO_4_ associated with Fe(PO_3_)_3_ at 750 °C. These intermediate phases are unstable and convert into α-Fe_2_O_3_, which is the final product of ferrihydrite transformation.

Thermal transformation of P-doped ferrihydrite to hematite is more complex than that of pure ferrihydrite. The DTA analysis of the FHYD-02 sample shows two distinct endotherms with the minima at 133 and 410 °C (Figure 5). This feature indicates that the dehydration/dehydroxylation process proceeds in two stages that are associated with mass loss of ca. 11 wt % and 21 wt %, respectively. The first effect is analogous to the dehydration of the surface, which is also observed for pure ferrihydrite (FH) at ca. 137 °C. The second thermal effect, a small endotherm at ca. 410 °C, is associated with the release of CO_2_ from the surface of partially substituted P-ferrihydrite (Figure S3). This is observed only for sample FHYD-02, and the reason is unknown. A broad exotherm at ca. 560 °C is probably related to the polymerization of iron phosphate groups [34].

The presence of P hinders the thermal transformation of FH to hematite. Instead of 460 °C as that for pure FH, the transformation of the FHYD-02 sample started above 500 °C. The formation of hematite proceeds through the formation of nanomaghemite γ-Fe_2_O_3_ as an intermediate phase. This is pronounced on the DTA curve by an exotherm at 660 °C (Figure 5). The poorly crystalline hematite was detected as a sole crystalline transformation product of FHYD-02 at 710 °C. A further increase in temperature results in the formation of small amounts of Fe(PO_3_)_3_ and rodolicoite FePO_4_, which associate with hematite. The admixture of Fe_7_(PO_4_)_6_ is also possible. Crystallization of these phases is marked by an exothermic peak on the DTA curve at 722 °C. The formation of iron phosphates, including FePO_4_, Fe(PO_3_)_3_, Fe_7_(PO_4_)_6_, and Fe_3_PO_7_, by the thermal treatment of various Fe-oxyhydroxides synthesized in the presence of phosphates was reported previously [34,59,60,61,62,63]. These intermediates (poorly crystalline hematite associated with various Fe-phosphates) gradually transform to hematite above 855 °C, and therefore, the XRD pattern at 1000 °C reveals hematite as the sole crystalline phase (Figure 5). The small amounts of P were probably partially incorporated into the hematite solid solution (as evidenced by broadening of XRD peaks, Figure S4) and partially solidified as amorphous phosphates (in small amounts not detectable by XRD).

**Figure 6:**
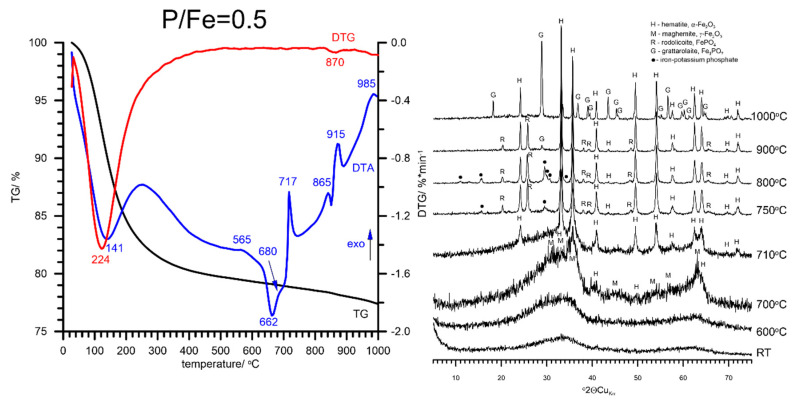
The results of thermal analysis and XRD patterns of P-doped synthetic ferrihydrite at the P/Fe molar ratio of 0.5. The formation of rodolicoite as an unstable intermediate at 717 °C is followed by transformation into grattarolaite Fe_3_PO_7_, which remains stable along with α-hematite up to 1000 °C. Note the formation of nanomaghemite γ-Fe_2_O_3_ at around 700 °C.

Thermal transformation of FHYD-05 begins with the dehydration process at a little higher temperature than that for pure FHYD and FHYD-02 (dehydration endotherm at ca. 141 °C instead of 130–133 °C). Moreover, the thermal pattern of the FHYD-05 sample does not show any distinct effects from the dehydroxylation process. This may indicate gradual dehydroxylation at a broad temperature range induced by P coverage. Above 680 °C, the P-doped synthetic ferrihydrite transforms into nanocrystalline maghemite (Figure 6). At ca. 710 °C, a poorly crystalline hematite is formed, associated with rodolicoite FePO_4_ and iron-potassium phosphate (observed in the temperature range of 750–800 °C). Above 800 °C, both these phosphates transform to more stable grattarolaite Fe_3_PO_7_, which remains stable along with hematite up to 1000 °C. On the basis of the phase diagram of the FePO_4_–Fe_2_O_3_ system by [63,64], the phase transformation at two exothermic effects without appreciable weight loss at ~717 °C and ~885 °C is most likely ascribed to the two solid-state phase transition of α to β and β to γ FePO_4_, as reported for quartz-like materials. At a high temperature range, the thermal transformation of FHYD-05 proceeds slightly differently from that of FHYD-02. The incorporation of P into hematite and the formation of distinct Fe-phosphate phases compete with each other. In the case of FHYD-02, there is not enough P to form distinct Fe-phosphates, and the formation of grattarolaite was not observed. However, in the case of FHYD-05, there is enough P to form a phosphate phase (high in Fe, though). This indicates the need for the presence of more P to form grattarolaite. Other parameters can also play a role that remains to be determined.

**Figure 7:**
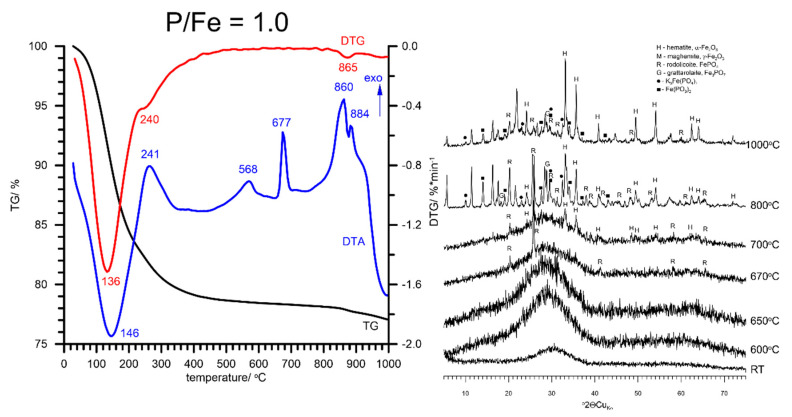
The results of thermal analysis and XRD patterns of P-doped synthetic ferrihydrite at the high P/Fe molar ratio of 1. Rodolicoite, which appears above 650 °C, is stable up to 1000 °C. This is associated with the formation of grattarolaite Fe_3_PO_7_ and other Fe-phosphates above 800 °C. All these phases, possibly partially precipitated from the melt, remain stable up to 1000 °C.

The TG/DTG/DTA curves of FHYD-10 are shown in Figure 7. The thermal transformation path for this high-P ferrihydrite is different from that for low-P samples described above. The position of the dehydration endotherm shifted to a higher temperature (ca. 146 °C). A similar behavior was previously noted for Si-ferrihydrites [24]. The associated mass loss of 10% corresponds to the presence of molecular water. A continuous weight loss of 12% between 150 and 800 °C is measured. Following the reports by Suber et al. [34], a broad exotherm observed at 568 °C can be attributed to the polymerization processes of iron phosphate groups. The next peaks are three exotherms at 677, 860 and 884 °C. The first product of FHYD-10 transformation is rodolicoite, which forms at ca. 670 °C. An XRD pattern at 700 °C shows the presence of hematite. Therefore, a sharp exothermic peak at 677 °C is attributable to the beginning of α-Fe_2_O_3_ crystallization. Above 700 °C, other phases are also formed, including Fe_3_PO_7_ (grattarolaite), Fe(PO_3_)_2_, and iron-potassium phosphate structurally resembling K_3_Fe(PO_4_)_2_. This mixture remains stable up to 1000 °C. It is associated with some amorphous material, possibly quenched melt, indicated by a distinctly elevated background on the XRD pattern between 10 and 35° 2Θ (Figure 7). A comparison of the position of major thermal effects apparent on DTA curves for pure and P-doped ferrihydrites is presented in Table S1.

**Figure 8:**
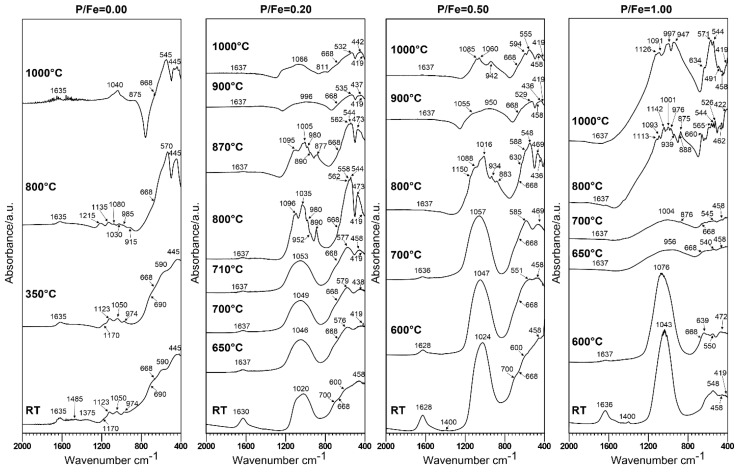
Comparison of background corrected FTIR spectra of P-doped ferrihydrites heated to various temperatures. RT—room temperature.

A comparison of background-corrected infrared spectra of P-doped ferrihydrites heated to various temperatures is presented in Figure 8. As indicated by the XRD analysis, heating of pure ferrihydrite transforms it into hematite α-Fe_2_O_3_. This is already manifested at 550 °C by bands typical of hematite at 440–470 and 545 cm^−1^ [37,64]. The absorption effects around 1635 cm^−1^ probably result from moisture adsorbed from the air during sample preparation. The appearance of the spectrum still slightly changes for a sample heated to 1000 °C, which is consistent with the XRD pattern. This indicates that, at 550 °C, a highly disordered hematite is formed, which recrystallizes at higher temperatures [24].

Heating of P-ferrihydrites leads to thermal transformations that are apparent on FTIR spectra at higher temperatures than for pure ferrihydrite. This is consistent with the changes observed on XRD patterns. The spectra of FHYD-02 and FHYD-05 samples remain practically unchanged up to 710 °C, and those for the FHYD-10 sample up to 600 °C. The position of a broad phosphate band shifts slightly toward the higher wavenumber (from 1024 to 1053 cm^−1^, from 1024 to 1057 cm^−1^, and from 1043 to 1076 cm^−1^ for FHYD-02, FHYD-05, and FHYD-10, respectively). The splitting of Fe–O bands in the range of 400–600 cm^−1^ may result from partial transformation into maghemite, as indicated by the XRD analysis. At 800 °C, the splitting of the wide phosphate band (1053–1076 cm^−1^) into many smaller ones is observed. The position of these bands varies slightly depending on the P content: ~1096, 1035–1005, 980–934, and 890–883 cm^−1^. At 900 °C, the splitting disappears, and the spectra of FHYD-02 and FHYD-05 samples show an identical, wide, and rounded band, derived mainly from rhodolicoite associated with hematite (as indicated by the XRD analysis), possibly along with amorphous phosphates. The widening of this broad phosphate band is attributed to the increasingly amorphous state [34], which is particularly well pronounced for ferrihydrites FHYD-02 and FHYD-05 heated to 900 °C and FHYD-10 heated to 650 and 700 °C. Moreover, absorption in the range of 473–469, 562–544, and 630–660 cm^−1^ is apparent. Some of these bands originate from hematite and others from different phases of iron phosphates, as the absorption maxima resulting from O–P–O bonds and from Fe–O bonds overlap in the range of 400–700 cm^−1^ [50]. For the final products of roasting at 1000 °C, the spectra of hematite are clear, but modified by the presence of phosphates. Although no additional phases other than hematite were detected by XRD in the product of FHYD-02 roasted at 1000 °C, the infrared absorption spectrum of this substance differs from that of hematite resulting from the transformation of pure ferrihydrite. A broad phosphate band in the range 900–1200 cm^−1^ is present, and the positions of the other bands are shifted slightly toward higher wavenumbers. The strongest effects from the presence of phosphates are marked on the spectra of FHYD-10. In the range of 700–1300 cm^−1^, there is a wide band with multiple splitting. This can be, at least partly, the evidence for the presence of amorphous phosphate glass. The presence of an amorphous substance is responsible for the elevated background observed on XRD patterns in the range 10–35° 2Θ, which is particularly apparent for the FHYD-10 sample. However, the presence of distinct bands at 1126, 1091, 997, and 947 cm^−1^ on FTIR spectra probably results from various Fe-phosphates, including grattarolaite, identified by XRD [63]. In the range of 400–700 cm^−1^, apart from the slightly shifted bands of hematite (634, 544, 458, and 419 cm^−1^), new bands appear at 571 and 491 cm^−1^. A clear decay of ferrihydrite structure above 600°C is observed for the FHYD-10 sample, which is more apparent on infrared spectra than on XRD curves. Rodolicoite is not stable in heated samples with intermediate P/Fe molar ratios of 0.20 or 0.50. However, at the highest P/Fe = 1.00, rodolicoite is still present in the final product of FHYD-10 heated up to 1000 °C (characteristic bands at 634, 571, and 491 cm^−1^), along with grattarolaite (1091 and 947 cm^−1^), hematite (544, 458, and 419 cm^−1^) and other phosphates. This indicates that, in the presence of elevated content of P, the thermal stability of rodolicoite and grattarolaite is extended to above 1000 °C. More detailed studies should be conducted in the future to explain this phenomenon.

In summary, comparison with previous work on the synthesis of Fe-phosphates [60,61,63] and the similarity of FTIR spectra of ferrihydrites doped with P with those of other Fe-phosphate minerals [55,58,62] indicate that phosphates are built into the structure of the mineral. The spectral analysis of the roasting product confirms the fact that the presence of P stabilizes the structure of ferrihydrite. Systematic shift of the main PO_4_ absorption band position on IR spectra for P-doped ferrihydrites is observed (Figure S5). The position of the band in the spectrum can be used to estimate the P content in ferrihydrite. The highest stability is obtained for FHYD-02 and FHYD-05. Their spectra are only slightly modified up to 800 °C. The FHYD-10 sample shows a slightly lower thermal stability, and its spectra are altered during roasting above 600 °C.

**Figure 9:**
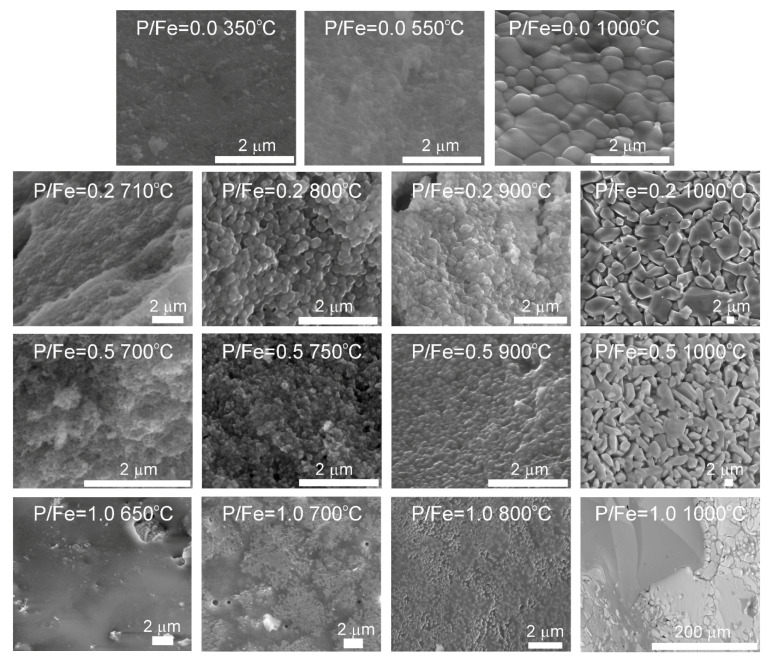
Comparison of morphologies of P-doped ferrihydrites heated to various temperatures and final products annealed at 1000 °C (SEM, secondary electron micrographs).

The selected SEM micrographs of P-doped ferrihydrites roasted to various temperatures are shown in Figure 9 and Figure S6. The particle shape and size evolve with P content as well as with temperature. FHYD-02 and FHYD-05 samples quenched at 650–900 °C are in the form of aggregates of fine particles of less than 1 μm in size, while FHYD-10 appears to be impregnated with solidified melt. The particles of thermally transformed FHYD-05 are smaller than that of FHYD-02. The increasing P content has a particularly strong effect on the morphology of the final products (heated to 1000 °C). Hematite particles resulting from the transformation of pure ferrihydrite are small (usually <1 μm in size), isometric, and form compact aggregates. On the other hand, the heating of P-doped ferrihydrite results in the formation of distinct, elongated grains of hematite up to 10 μm in size that form porous random aggregates. Their morphology resembles that of hematite resulting from the roasting of Si-doped ferrihydrite at a higher Si/Fe ratio [24]. The FHYD-10 sample converts thermally into a heterogeneous mixture of Fe_2_O_3_ and iron-phosphate nanoparticles submerged in phosphate glass.

**Figure 10:**
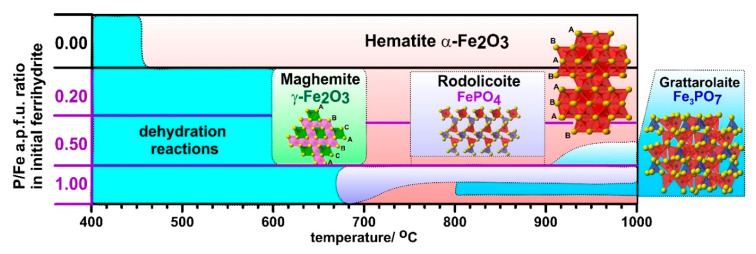
Schematic presentation of temperature ranges of phase stability during thermal transformations of P-doped ferrihydrite.

The stability temperatures for P-doped ferrihydrites are summarized in Figure 10. During the thermal transformation of P-ferrihydrite, the formation of rodolicoite FePO_4_ associated with grattarolaite Fe_3_PO_7_ at higher temperatures (above 900 °C) is observed, although the presence of other iron phosphates cannot be excluded. These results also indicate that the formation of rodolicoite and grattarolaite is possible by heating P-ferrihydrite to high temperatures (between 700 and 1000 °C), and can be controlled by both P content and temperature.

## 4. Summary and Conclusions

Coprecipitation of P with ferrihydrite results in the formation of P-doped 2-line ferrihydrite. According to [45], phosphate ions and Fe(III) in aqueous suspensions can form both ternary complexes (most likely as (≡Fe–O)_2_–PO_2_–Fe due to the nature of positive charge at the surfaces) and prenucleation clusters that may further condense to precipitates of iron phosphates. With increasing P sorption loading, the sorption mechanism transits from a bidentate-binuclear surface complexation to an unidentified ternary complexation and then to precipitation of amorphous FePO_4_. These may be the likely mechanisms of precipitation during the synthesis of P-doped ferrihydrite at increasing concentrations of P. At a high concentration of P, the crystalline structure of ferrihydrite revealed by XRD is strongly affected. Thus, it may be more appropriate to use the term “ferrihydrite-like phase rich in P” for the synthesized product with a high P/Fe molar ratio of 1.

The presence of P coprecipitated with ferrihydrites strongly affects their thermal conversion paths. In all cases, the product of heating of ferrihydrite up to 1000 °C contains hematite. Phosphate significantly inhibits the transformation process. A similar effect was observed for arsenates [23]. The temperature of thermal transformation increases from below 550 °C to between 710–750 °C. The formation of intermediate phases, namely maghemite and Fe-phosphates, is observed. At a higher P/Fe ratio, the final product consists of a mixture of hematite and crystalline as well as amorphous phosphates. This includes rodolicoite FePO_4_ and grattarolaite Fe_3_PO_7_.

The results presented in this paper show that the P content greatly affects the thermal stability and transformation temperature of not only ferrihydrite but also rodolicoite. Our results are consistent with the phase diagram of the FePO_4_–Fe_3_PO_7_–Fe_2_O_3_ system proposed by Zhang et al. [65,66]. This diagram presents the relative stability of rodolicoite compared to that of grattarolaite. During the thermal transformation of FHYD-02, rodolicoite accompanies hematite in the temperature range of 750–900 °C and then undergoes thermal decomposition and disappears from XRD diffraction curves at 1000 °C. At higher P concentration (P/Fe molar ratio of 0.5), rodolicoite undergoes thermal transformation into grattarolaite above 900 °C; thus, grattarolaite and hematite remain the final products of this process. At a higher P/Fe molar ratio, a similar thermal transformation causes the crystallization of rodolicoite even before that of hematite. Rodolicoite is apparent on XRD patterns since 670 °C and remains up to 1000 °C despite partial transformation into grattarolaite and the formation of other iron-phosphate phases (possibly through partial melting) and hematite.

## Supplementary Materials

The following are available online at https://www.mdpi.com/1996-1944/13/18/4113/s1, Figure S1: Comparison of EDS spectra of pure and P-doped ferrihydrites, Figure S2: Quadrupole mass spectrometry (QMS) signal recording a release of SO_2_ between 600 and 800 °C, Figure S3: Quadrupole mass spectrometry (QMS) signal recording a release of H_2_O and CO_2_ upon heating of P-doped ferrihydrite, Figure S4: Fragment of XRD pattern of hematite resulting from heating of sample FHYD-0.2 to 900 °C (narrower, blue pattern) and to 1000 °C (broader, red pattern) resulting probably from incorporation of P into hematite solid solution, Figure S5: **A**. Systematic shift of the main PO4 absorption band position on IR spectra for P-doped ferrihydrites. The position of the band in the spectrum can be used to estimate the P content in ferrihydrite. **B**. The effect of temperature on the shift of absorption bands originating from PO4 is almost identical for P-poor and P-rich ferrihydrites, Figure S6: SEM image and EDS spectra of products of P-rich ferrihydrite (FHYD-1.0) heating to 800 °C (**A**) and to 1000 °C (**B**), Table S1: Comparison of the position of major thermal effects apparent on DTA curves for pure and P-doped ferrihydrites.
