# Supplementary material for: Thermal Stability and Decomposition Products of P-Doped Ferrihydrite
Source: Materials (Basel). 2020 Sep 16;13(18):4113. doi: 10.3390/ma13184113 (PMC7560356; doi:10.3390/ma13184113)
Supplement: Supplementary file 1 [file materials-13-04113-s001.pdf]

materials

Supplementary Information

Thermal Stability and Decomposition Products of P-Doped Ferrihydrite

Gabriela Pieczara [1], Maciej Manecki [1,*], Grzegorz Rzepa [1], Olaf Borkiewicz [2] and Adam Gaweł [1]

[1] Faculty of Geology, Geophysics and Environmental Protection, AGH-University of Science and Technology, al. Mickiewicza 30, 30-059 Kraków, Poland; gabriela.pieczara@gmail.com (G.P.); gprzepa@cyf-kr.edu.pl (G.R.); agawel@agh.edu.pl (A.G.)

[2] X-ray Science Division, Advanced Photon Source, Argonne National Laboratory, Argonne, Illinois 60439, USA; borkiewicz@aps.anl.gov

* Correspondence: gpmmanec@cyf-kr.edu.pl; Tel.: +48-604-427-198

Received: 18 August 2020; Accepted: 14 September 2020; Published: date

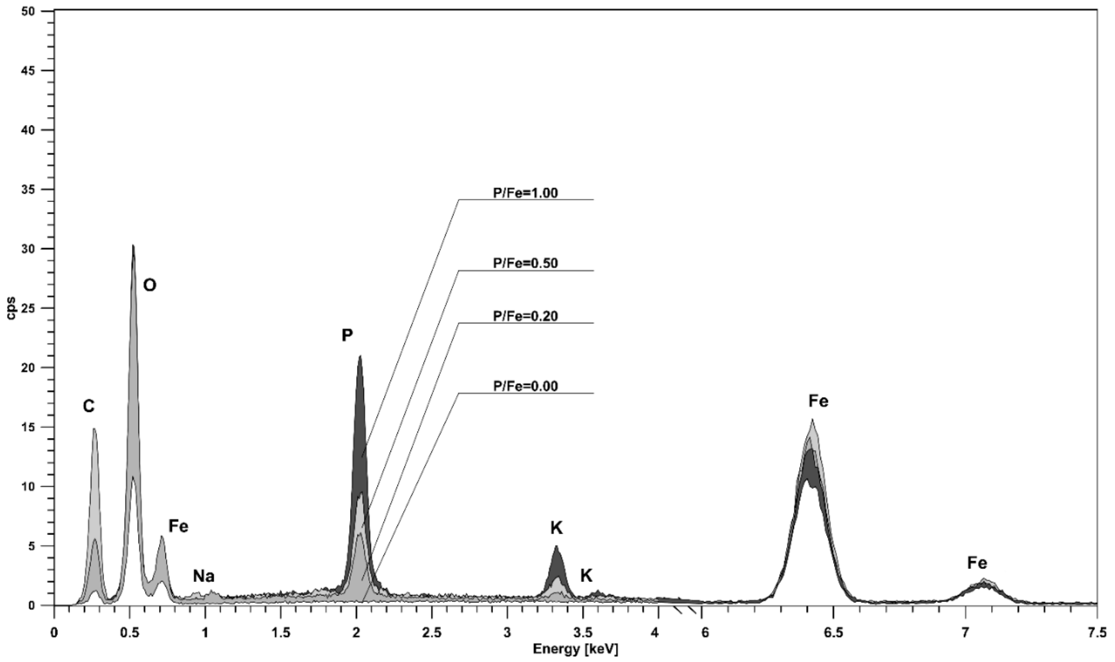

Figure S1. Comparison of EDS spectra of pure and P-doped ferrihydrites.

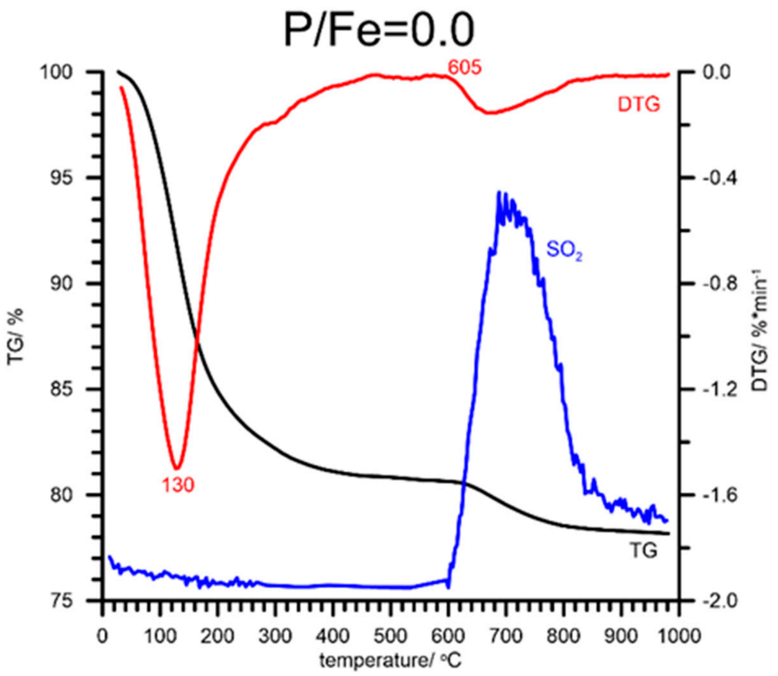

Figure S2. Quadrupole mass spectrometry (QMS) signal recording a release of SO_2 between 600 and 800 °C.

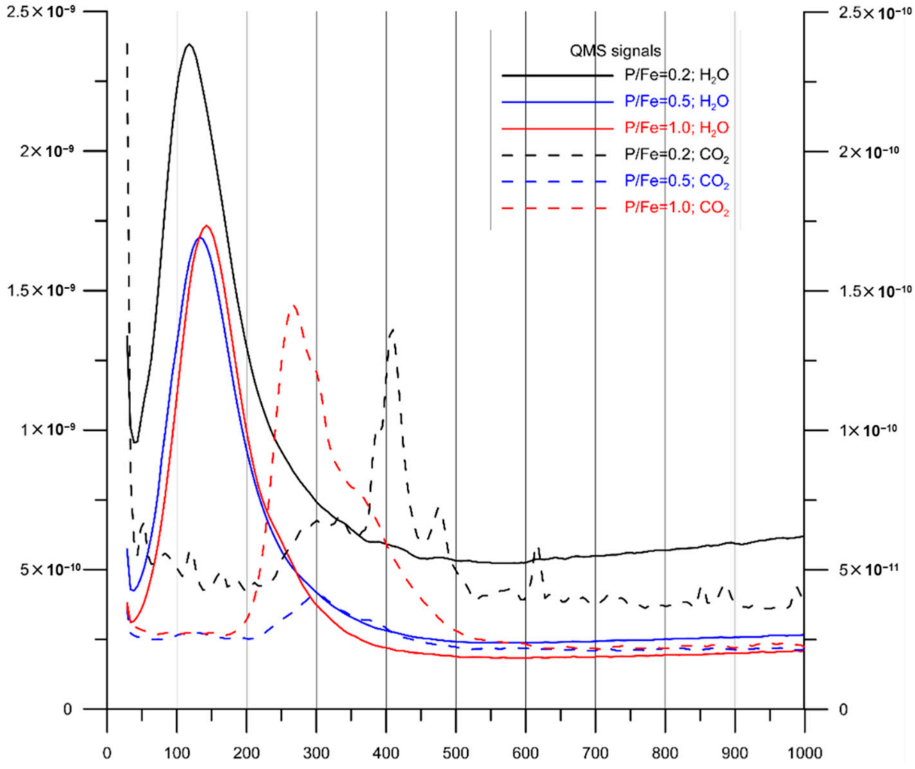

Figure S3. Quadrupole mass spectrometry (QMS) signal recording a release of H_2O and CO_2 upon heating of P-doped ferrihydrite.

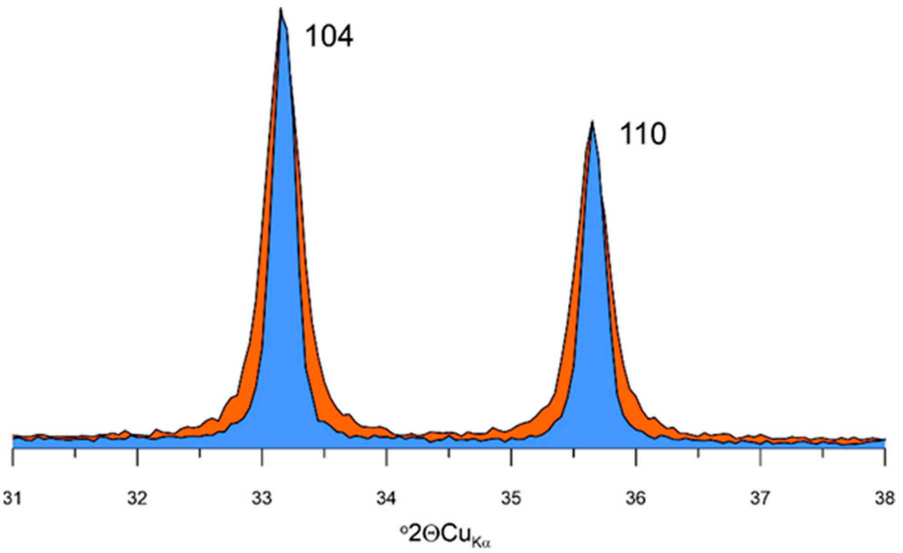

Figure S4. Fragment of XRD pattern of hematite resulting from heating of sample FHYD-0.2 to 900 °C (narrower, blue pattern) and to 1000 °C (broader, red pattern) resulting probably from incorporation of P into hematite solid solution.

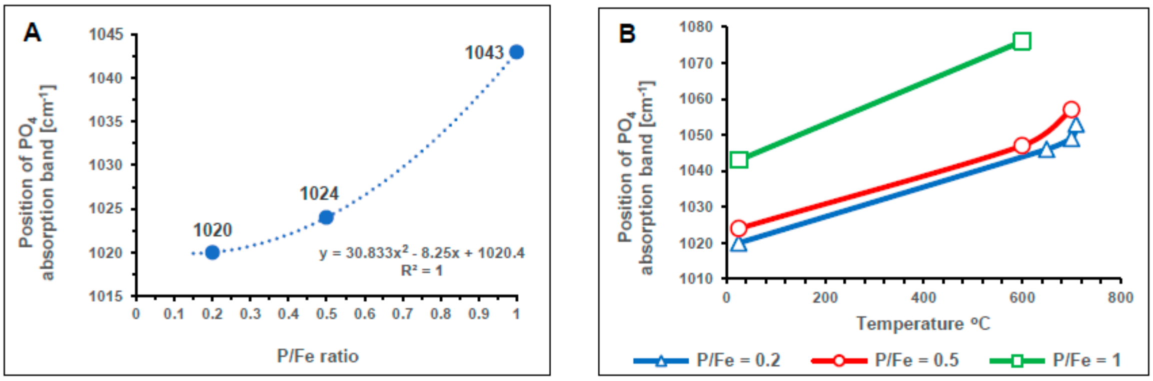

Figure S5. A. Systematic shift of the main PO4 absorption band position on IR spectra for P-doped ferrihydrites. The position of the band in the spectrum can be used to estimate the P content in ferrihydrite. **B**. The effect of temperature on the shift of absorption bands originating from PO4 is almost identical for P-poor and P-rich ferrihydrites.

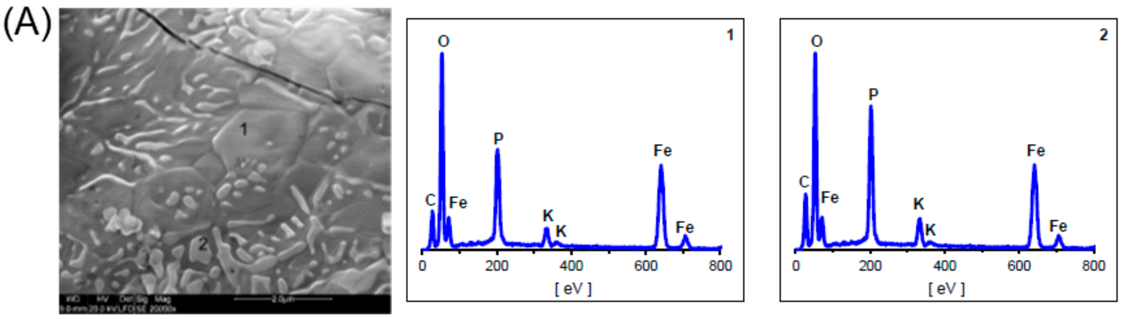

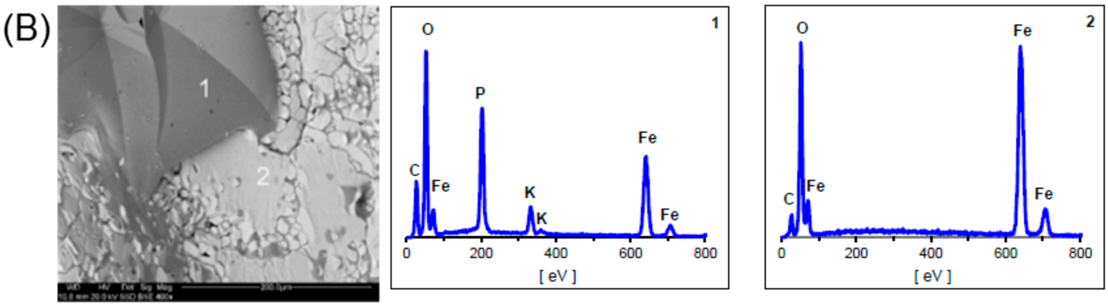

Figure S6. SEM image and EDS spectra of products of P-rich ferrihydrite (FHYD-1.0) heating to 800 °C (**A**) and to 1000 °C (**B**).

Table S1. Comparison of the position of major thermal effects apparent on DTA curves for pure and P-doped ferrihydrites.

	**P/Fe**	**0.0**	**0.2**	**0.5**	**1.0**
Dominating processes responsible for thermal effect		Temperature °C			
Removal of surface moisture	endo	137	133	141	146
Ferrihydrite collapse	exo	460	558	565	568
Formation of nanomaghemite	exo		658	680	677
Formation of Fe-phosphates and rodolicoite	exo		722	717	
Formation of hematite and transformation of Fe-phosphates	exo		855	865	860
Transformation of rodolicoite and formation of grattarolaite	exo			915	884

© 2020 by the authors. Submitted for possible open access publication under the terms and conditions of the Creative Commons Attribution (CC BY) license (http://creativecommons.org/licenses/by/4.0/).
